# Regional STEMI Program Historical Mortality Rates in Maine, USA

**DOI:** 10.5811/westjem.48505

**Published:** 2025-07-18

**Authors:** Olivia Pearson, Sara Kovacs, Rachel Crowe, Thomas Ryan, Colin Phillips

**Affiliations:** MaineHealth Maine Medical Center, Department, Portland, Maine

## Introduction

Without timely reperfusion therapy, 1-month mortality rates for ST-elevation myocardial infarction (STEMI) range from 10–25%. The addition of medical therapies has improved mortality rates to 1–8% (ISIS II, MORACS). This mortality rate is further reduced with percutaneous coronary intervention (Keeley). The chain of survival from patient recognition to proper and timely treatment requires ongoing assessment and monitoring. Given geographic constraints, patients presenting with STEMI in Maine are treated with both fibrinolysis and primary PCI depending on where they are first identified. The regional AMI-PERFUSE program represents an ongoing effort to provide optimal care for patients presenting with STEMI. Here we report historical mortality rates of patients presenting with STEMI based on treatment modality.

## Methods

Utilizing historical databases of consecutive unselected patients treated for STEMI, we assessed and organized outcomes at the patient level. Each STEMI case was adjudicated to ensure proper classification. Treatment modalities included PCI, fibrinolysis with PCI, fibrinolysis alone, or no reperfusion therapy. Surgical revascularization was not specified within the database. Percentages and 2-tailed Z-test were used to analyze the data.

## Results

**Figure 1:**
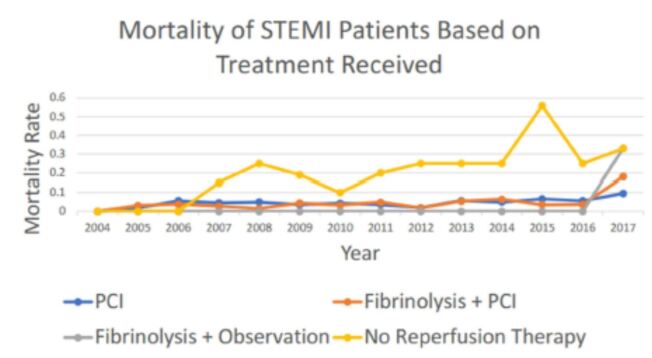
Longitudinal mortality rates of STEMI patients according to treatment modality received.

From 2004–2017, 5,945 patients presented with STEMI and were analyzed (Table 1). There were 317 deaths (5.3%), with a 2017 aberration of 59 total deaths. 195 patients (3.2%) whose treatment was not classified were excluded from the analysis. Overall, in-hospital mortality rate following STEMI presentation was 5.3% for those receiving treatment. Between 2004–2016, mortality rates peaked at 7% for patients treated with primary PCI (n=3,243) and fibrinolysiswith PCI (n=2,230; see Figure 1). There was no significant difference in MaineHealth Maine Medical Center, Department, Portland, Maine overall mortality rates between the two populations (4.8% vs. 4.7%, p=0.84). Mortality rates between patients treated with PCI (54.5%, n= 3243) vs. fibrinolysis alone (0.9%, n=107) and between those treated with fibrinolysis with PCI (37.5%, n = 2230) vs. fibrinolysis alone (0.9%, n=107) were also not significantly different (p=0.06 and p=0.07, respectively). The in-hospital mortality rates for all three treatment modalities were significantly decreased from those who did not receive reperfusion therapy (23.5%, p< 0.01 for all comparisons, n=170).

## Conclusions

Over 14 years of available historical data from a regional STEMI database, in-hospital mortality rates for patients varied along a narrow margin and did not differ based on treatment modality. Regardless of treatment, mortality was significantly lower compared with those who received no reperfusion therapy.

## Figures and Tables

**Table 1:** Comparison of mortality rates between STEMI treatment modalities and associated significance of outcomes.

Treatment	Mortality Rate	Z-Test Statistic	Significance in Over Mortality Rates
PCI vs. Lytics + PCI	4.78% vs. 4.66%	0.1983	P = 0.84
PCI v. Lytics + Observation	4.78% vs. 0.03%	1.857	P = 0.06
PCI vs. No reperfusion therapy	4.78% vs. 23.5%	−10.27	P < 0.001
Lytics + PCI vs. Lytics + Observation	4.78% vs. 0.03%	1.819	P = 0.07
Lytic + PCI vs. No reperfusion therapy	4.78% vs. 23.5%	−9.984	P < 0.001
Lytics + Observation + No reperfusion therapy	0.03% vs. 23.5%	−5.156	P < 0.001

*STEMI*, ST-elevation myocardial infarction, *PCI*, percutaneous coronary intervention, *Lytics*, thrombolytic therapy.

